# Complete Genome Sequence of Plant Growth-Promoting Rhizobacterium Pseudomonas protegens SN15-2

**DOI:** 10.1128/MRA.01548-19

**Published:** 2020-04-16

**Authors:** Xiaobing Wang, Mengfei Wang, Danyan Tang, Wei Wang

**Affiliations:** a State Key Laboratory of Bioreactor Engineering, East China University of Science and Technology, Shanghai, China; University of Arizona

## Abstract

We report the complete genome sequence of Pseudomonas protegens SN15-2, which was isolated from the rhizosphere of tomato roots in Shanghai, China, and can be used in the biological control of phytopathogens. This annotated version will be the basis for upcoming genomic studies.

## ANNOUNCEMENT

Pseudomonas protegens SN15-2, formerly part of the Pseudomonas fluorescens complex (1), was isolated from tomato roots in Shanghai, China, and its metabolites have been identified as being effective in inhibiting the growth of Ralstonia solanacearum (2). The sequencing of the P. protegens SN15-2 genome will provide basic information for future research on molecular regulation in SN15-2.

A single colony of SN15-2 was inoculated into nutrient broth and cultured for 24 h at 28°C, and the cells were collected for sequencing. The SN15-2 genome was determined using the PacBio single-molecule real-time (SMRT) sequencing platform by Frasergen (China). In the PacBio sequencing, a total of 99,553 subreads (mean length of 5,975 bp) were *de novo* assembled with the Hierarchical Genome Assembly Process (HGAP) (3). Default parameters were used for all software unless otherwise specified. A total of 594.88 Mb of data were produced. Based on the sequencing data, a complete circular chromosome was successfully assembled. The complete genome of P. protegens SN15-2 contains one circular chromosome of 7.08 Mb, with a G+C content of 63.3%. The SN15-2 complete genome was annotated using the NCBI Prokaryotic Genome Annotation Pipeline (PGAP) (4) and was predicted to contain 6,375 genes, including 6,238 protein-coding genes, 16 rRNAs, 70 tRNAs, 4 noncoding RNAs, and 47 pseudogenes.

The SN15-2 genome is highly similar to the genomes of P. protegens H78 (GenBank accession number CP013184; 98.86% identical, based on average nucleotide identity) (5) and CHA0 (GenBank accession number LS999205; 98.81% identical, based on average nucleotide identity) (6). The P. protegens species can produce a specific spectrum of antibiotic compounds, including pyoluteorin, 2,4-diacetylphloroglucinol, and pyrrolnitrin, and has been identified as an independent taxon well separated from other pseudomonads (1).

### Data availability.

The genome sequence of P. protegens SN15-2 was deposited in GenBank under BioProject accession number PRJNA557545 and accession number CP043179. The raw reads were deposited in the NCBI Sequence Read Archive (SRA) under accession number SRR10728566.
